# Impact of changes to national guidelines on hypertension-related workload: an interrupted time series analysis in English primary care

**DOI:** 10.3399/bjgp21X714281

**Published:** 2020-03-23

**Authors:** Sarah L Lay-Flurrie, James P Sheppard, Richard J Stevens, Christian Mallen, Carl Heneghan, FD Richard Hobbs, Bryan Williams, Jonathan Mant, Richard J McManus

**Affiliations:** Nuffield Department of Primary Care Health Sciences, University of Oxford, Oxford.; Nuffield Department of Primary Care Health Sciences, University of Oxford, Oxford.; Nuffield Department of Primary Care Health Sciences, University of Oxford, Oxford.; School for Primary, Community and Social Care, Keele University, Staffordshire.; Nuffield Department of Primary Care Health Sciences, University of Oxford, Oxford.; Nuffield Department of Primary Care Health Sciences, University of Oxford, Oxford.; National Institute for Health Research (NIHR) University College London Hospitals Biomedical Research Centre, Institute of Cardiovascular Science, University College London, London.; Primary Care Unit, Department of Public Health & Primary Care, University of Cambridge, Cambridge.; Nuffield Department of Primary Care Health Sciences, University of Oxford, Oxford.

**Keywords:** blood pressure, consultation, general practice, guideline, hypertension, workload

## Abstract

**Background:**

In 2011, National Institute for Health and Care Excellence (NICE) guidelines recommended the routine use of out-of-office blood pressure (BP) monitoring for the diagnosis of hypertension. These changes were predicted to reduce unnecessary treatment costs and workload associated with misdiagnosis.

**Aim:**

To assess the impact of guideline change on rates of hypertension-related consultation in general practice.

**Design and setting:**

A retrospective open cohort study in adults registered with English general practices contributing to the Clinical Practice Research Datalink between 1 April 2006 and 31 March 2017.

**Method:**

The primary outcome was the rate of face-to-face, telephone, and home visit consultations related to hypertension with a GP or nurse. Age- and sex-standardised rates were analysed using interrupted time-series analysis.

**Results:**

In 3 937 191 adults (median follow-up 4.2 years) there were 12 253 836 hypertension-related consultations. The rate of hypertension-related consultation was 71.0 per 100 person–years (95% confidence interval [CI] = 67.8 to 74.2) in April 2006, which remained flat before 2011. The introduction of the NICE hypertension guideline in 2011 was associated with a change in yearly trend (change in trend −3.60 per 100 person–years, 95% CI = −5.12 to −2.09). The rate of consultation subsequently decreased to 59.2 per 100 person–years (95% CI = 56.5 to 61.8) in March 2017. These changes occurred around the time of diagnosis, and persisted when accounting for wider trends in all consultations.

**Conclusion:**

Hypertension-related workload has declined in the last decade, in association with guideline changes. This is due to changes in workload at the time of diagnosis, rather than reductions in misdiagnosis.

## INTRODUCTION

Primary care workload in England increased by 16% between 2007 and 2014, with primary care appearing to reach saturation point.^1^ A key component of this workload is the diagnosis and management of long-term chronic conditions. In particular, hypertension (high blood pressure) affects 14% of the population in the UK,^2^ and is a leading risk factor for stroke and coronary heart disease.^3^ Identifying and treating hypertension reduces the risk of stroke and coronary heart disease,^4^ and is cost-effective.^5^

The 2011 National Institute for Health and Care Excellence (NICE) hypertension guideline introduced recommendations for the diagnosis of hypertension that included the use of out-of-office measurement for confirmation of an initially raised clinic blood pressure (BP).^5^ This change was in response to concerns that using clinic BP may result in approximately 25% of individuals being misclassified due to white coat hypertension.^6^ The changes were predicted to reduce workload due to a reduction in the unnecessary treatment of white coat hypertension.^5^

The authors have already examined the association between these guideline changes and clinical outcomes,^7^ demonstrating that changes to guidelines were associated with a levelling-off in the downward trend of hypertension incidence and no change in the rate of cardiovascular events.^7^ In the current study, the authors aimed to examine trends in rates of hypertension-related workload in general practice from April 2006 to March 2017 in England. The authors further aimed to test whether the introduction of the NICE hypertension guideline in 2011 was associated with a change in these trends.

## METHOD

### Study design

The methods used for this study have been fully described previously;^7^ this was a retrospective open cohort study of adults (aged ≥18 years) registered at English general practices contributing to the Clinical Practice Research Datalink (CPRD) between 1 April 2006 and 31 March 2017. Patients were included if their records were acceptable for research purposes (data defined by CPRD as ‘up to standard’) and eligible for data linkage, and they entered the study on the date they met all eligibility criteria. Patients were excluded if they had a history of hypertension before study entry, but were not excluded if they developed hypertension during follow-up.

**Table:** How this fits in

Recommendations for the use of out-of-office blood pressure (BP) measurement for the diagnosis of hypertension in the 2011 National Institute for Health and Care Excellence hypertension guideline were predicted to reduce general practice workload. This analysis shows that these changes were associated with a reduction in hypertension-related workload, in particular around the time of diagnosis. Practitioners are likely to benefit from time savings when using out-of-office BP measurement for diagnosis and treatment titration.

### Outcomes

The primary outcome was the rate of hypertension-related general practice consultation. Consultations and staff roles in CPRD were grouped into types, as previously.^1^ The authors’ primary analysis concerned face-to-face, telephone, or home visit consultations with a GP or nurse. A consultation was defined as hypertension-related if it included a clinical code for the diagnosis or management of hypertension, a recording of BP, or a prescription for antihypertensive medication (see Supplementary Appendix S1). The authors studied total hypertension-related consultation time (total length of hypertension-related consultations in minutes) as a secondary outcome. In post-hoc sensitivity analyses, the authors excluded consultations containing only a BP measurement.

Negative controls were used to determine whether changes in hypertension-related consultation rates were plausibly due to changes in guidance or other factors. These were asthma-related consultations (including a clinical code for asthma diagnosis or monitoring, or a prescription for asthma-related medications) and all consultations, regardless of the presenting condition. Asthma was chosen because it is primarily managed in primary care, similar to hypertension, but has a different pathophysiology, and completely different diagnosis and treatment pathways. The activities carried out to manage asthma are therefore unlikely to be affected by changes to hypertension guidelines.

### Statistical analysis

Crude and standardised rates were calculated stratified by age (18–24, 25–44, 45–54, 55–64, 65–74, 75–84, and >85 years) and sex in each month. Rates were standardised to the English national population standard in 2015. The authors conducted analyses stratified by consultation type and staff role, and subgroup analysis in patients with/without hypertension from March 2007 onwards (allowing 1 year for incident hypertension cases to develop). In post-hoc analyses, the authors examined consultation rates relative to the time of diagnosis of hypertension:

- within 6 months before diagnosis;
- >6 months before diagnosis;
- within 12 months after diagnosis; and
- >12 months after diagnosis.

Standardised rates were modelled using interrupted time series analysis (ITSA) with Newey-West standard errors.^8^ The authors assessed whether the introduction of the NICE hypertension guideline in 2011 was associated with a step change in consultation rates or a change in trend by interrupting the time series between 1 April 2011 and 31 March 2012. Analyses were weighted according to the total person–years of observation contributing to each monthly rate. Lag terms (up to 12 months) were included in sensitivity analyses. Analysis was conducted using Stata (version 14).

## RESULTS

In total 3 937 191 patients were eligible for inclusion in the study cohort (see Supplementary Figure S1), totalling 19 088 414 person–years of follow-up (median follow-up 4.2 years, interquartile range [IQR] 1.6–8.0). The characteristics of the cohort are given in Table 1. There were 12 253 836 hypertension-related consultations across the study period, or an average of 0.64 consultations per person, per year. Of these, 67.8% were with a GP and 97.0% were face-to-face consultations. The majority (86.8%) of consultations included a clinic BP measurement, and an antihypertensive prescription was issued in 21.3% of consultations (see Supplementary Table S1).

**Table 1.** Baseline study characteristics, N = 3 937 191

**Variable**	**%^a^**
Age, years, median (IQR)	36 (26–50)

Sex, male	49.0

Median follow-up, years	4.2

**Ethnicity**	
White	47.6
Non-White	9.0
Unknown	43.4

Prior MI or stroke	1.6

Prior CVD (MI, stroke, or other)	4.9

**IMD quintile**	
1 (least deprived)	22.1
2	22.1
3	19.9
4	20.6
5 (most deprived)	15.2
Unknown	0.1

a Unless otherwise stated. CVD = cardiovascular disease. IMD = Index of Multiple Deprivation. IQR = interquartile range. MI = myocardial infarction.

### Rate of hypertension-related consultation

**Figure 1.**
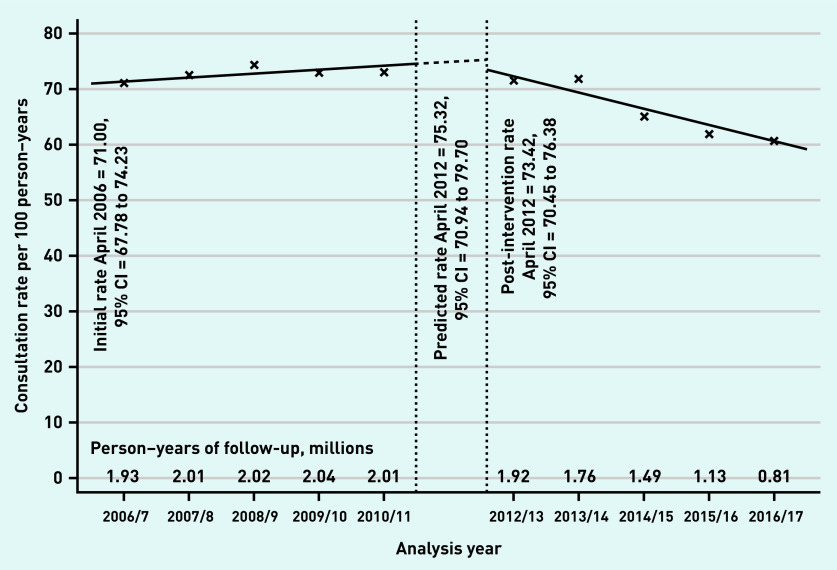
**Age- and sex-standardised rate of hypertension-related consultations (face-to-face, telephone, or home visit consultations with a GP or nurse per 100 person–years between April 2006 and March 2017, with interruption between April 2011 and March 2012). Fitted lines produced using interrupted time-series analysis on monthly adjusted rates.**

The crude rate of consultation (per 100 person–years) was notably higher in women of younger age compared to men, but increased with age in both men and women (see Supplementary Table S2). The standardised rate of hypertension-related consultation decreased over the study period from 71.0 per 100 person–years (95% confidence interval [CI] = 67.8 to 74.2) in April 2006 to 59.2 per 100 person–years (95% CI = 56.5 to 61.8) in March 2017 (Figure 1, Table 2). The introduction of the NICE hypertension guideline in 2011 was not associated with a significant change in the consultation rate level (change in rate −1.90, 95% CI = −7.20 to 3.39) but was associated with a change in the yearly trend (change in trend −3.60, 95% CI = −5.12 to −2.09). When excluding consultations containing only a BP measurement the rate of hypertension-related consultation was considerably lower, but changes to guidelines in 2011 were associated with both a change in the consultation rate level and trend (see Supplementary Table S3). When considering lag terms results were unchanged.

**Table 2.** Interrupted time-series analysis of age- and sex-standardised rates of hypertension-related consultation^a^

	**Estimate per 100 person–years**	**95% CI**
**Initial rate, April 2006**	71.00	67.78 to 74.23
**Initial trend per year, April 2006–March 2011**	0.71	−0.43 to 1.84
**Predicted rate, April 2012**	75.32	70.94 to 79.70
**Post-intervention rate, April 2012**	73.42	70.45 to 76.38
**Post-intervention trend per year, April 2012–March 2017**	−2.89	−3.89 to −1.90
**Change in rate**	−1.90	−7.20 to 3.39
**Change in trend**	−3.60	−5.12 to −2.09

a face-to-face, telephone, or home visit consultations with a GP or nurse per 100 person–years between April 2006 and March 2017, with interruption between April 2011 and March 2012.

**Figure 2.**
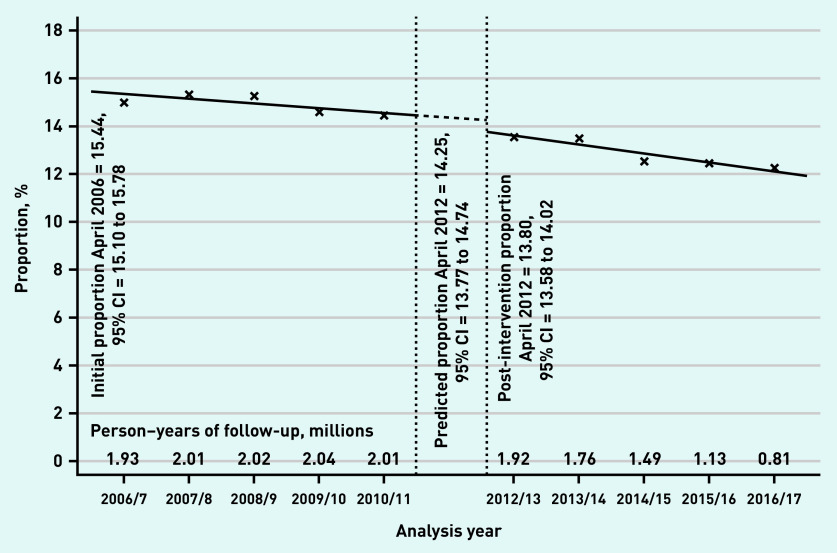
**Age- and sex-standardised rate of consultations related to hypertension, as a proportion of all consultations between April 2006 and March 2017, with interruption between April 2011 and March 2012. Fitted lines produced using interrupted time-series analysis on monthly adjusted rates.**

Analyses of asthma-related consultation and all consultations showed similar patterns — namely, no trend between 2006 and 2011 was followed by a downward trend until 2017 (see Supplementary Table S4, and Supplementary Figures S2 and S3). When the authors examined the rate of hypertension-related consultations as a proportion of all consultations they found that hypertension-related consultations accounted for 15.4% of all consultations in April 2006, decreasing to 13.8% in April 2012 and 11.9% in March 2017 (Figure 2). Guideline change in 2011 was associated with an acceleration of the downward trend (see Supplementary Table S5). Although the rate of hypertension-related consultation was stable pre-2011, the rate of all consultations was increasing. After 2011, the rate of hypertension-related consultation decreased at a faster pace than the rate of all consultations.

#### Stratified by hypertensive status

**Figure 3.**
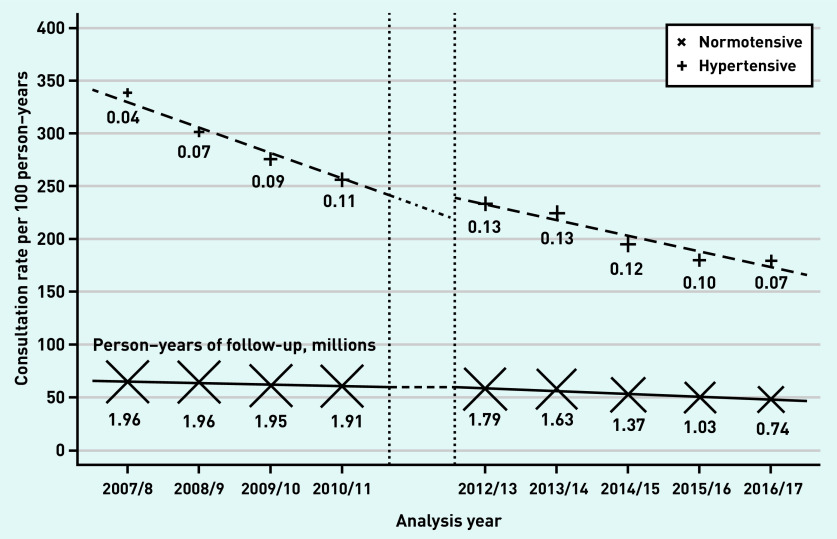
**Age- and sex-standardised rates of hypertension-related consultations in normotensive and hypertensive patients between April 2007 and March 2017, with interruption between April 2011 and March 2012. Fitted lines produced using interrupted time-series analysis on monthly adjusted rates.**

Stratified analyses demonstrated that the rate of hypertension-related consultation was significantly higher in hypertensive compared to normotensive patients (Figure 3, Supplementary Table S6). In normotensive patients, the change in guidance in 2011 was not associated with changes in consultation rate level or trend. In patients with hypertension, the rate of consultation fell from 341 per 100 person–years in April 2007 (95% CI = 326 to 357) to 166 in March 2017 (95% CI = 158 to 174), with a slowing of this downward trend after 2011. Consultation rates were highest in the diagnostic and initial treatment phases across the study period (see Supplementary Figure S4). The guideline changes were only associated with changes in trend (from no trend to downward trend) in the 6 months before diagnosis (change in trend −19.8, 95% CI = −36.6 to −3.0) and 12 months after diagnosis (change in trend −16.0, 95% CI = −23.7 to −8.3) (see Supplementary Table S7).

#### Stratified by consultation type and staff role

Observed patterns in consultation rates were driven by face-to-face consultations, which were unchanged between 2006 and 2011, and decreased year-on-year between 2012 and 2017 for both GPs and nurses (change in trend in 2011–2012 −2.28, 95% CI = −3.25 to −1.32) (see Supplementary Table S8). The rates of home visit consultation with a GP or nurse and telephone consultation with a nurse were unchanged across the entire study period. Conversely, the rate of telephone consultation with a GP increased between 2006 and 2011, and increased at a greater rate after the change in guidance in 2011.

### Rate of hypertension-related consultation time

**Figure 4.**
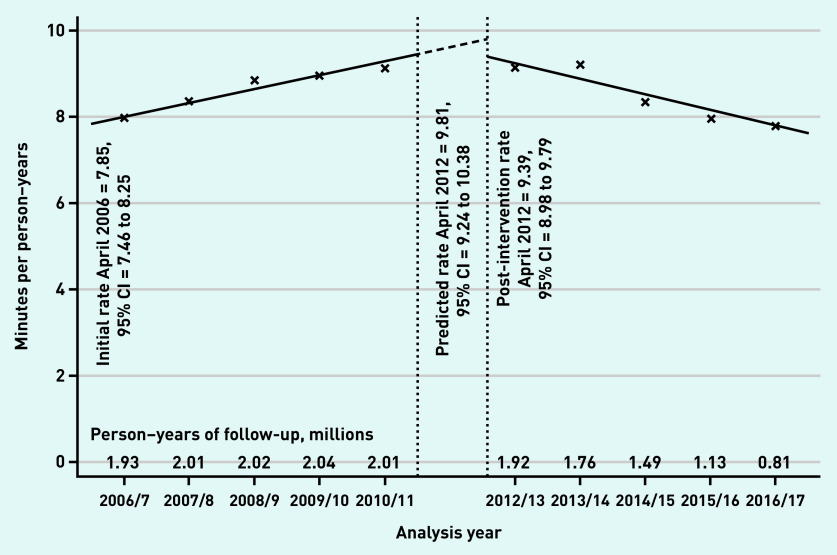
**Age- and sex-standardised rate of hypertension-related consultation time (minutes per person–year in face-to-face, telephone, or visit consultations with a GP or nurse per person–year between April 2006 and March 2017, with interruption between April 2011 and March 2012).**

Hypertension-related consultations accounted for 2.53 million hours of clinical time, equivalent to 7.94 minutes of consultation per person, per year on average. Results for consultation time mirrored those of consultation rates (Figure 4, Supplementary Table S9).

## DISCUSSION

### Summary

The rate of hypertension-related general practice consultation in England was stable between 2006 and 2010 and then fell between 2011 and 2017. This reduction was concurrent with similar changes in trend in the rate of asthma-related and all-cause consultations, suggesting that the new downward trend was driven in part by wider system- or population-level changes. However, changes in hypertension-related workload were relatively greater than changes in overall all-cause consultation rates and occurred primarily around the time of diagnosis, indicating that these changes may be plausibly associated with the guideline change in 2011. Similar patterns were observed for average consulting time.

### Strengths and limitations

This was a large scale analysis of data known to be representative of the UK population.^9^ Hence, the authors have been able to estimate rates with the precision required to detect meaningful differences in outcomes. The use of standardised rates further increases the likelihood that the results are applicable to the wider population.

A consultation in CPRD represents a distinct opening of a patient’s electronic healthcare record. This may occur to document a consultation or for administrative purposes. The authors grouped consultations by type to consider patient-facing clinical workload with a GP or nurse only, but some consultations may have been misclassified. However, the rate of consultation observed for normotensives is consistent with Quality and Outcomes Framework guidelines for BP to be measured every 5 years,^10^ indicating that the results have face validity.

The authors’ definition of hypertension-related activity did not include codes specifically related to cardiovascular disease (CVD) risk or prevention since, in England, guidelines for CVD prevention (largely related to statin prescription in relation to CVD risk) are separate to those for hypertension.^11^ Nevertheless, the authors’ inclusion of codes for any BP measurement is likely to have captured many consultations considering CVD risk more generally. Due to this inclusive definition, some consultations may have been misclassified (for example, the use of calcium channel blockers in Raynaud’s phenomenon), but these would not have been expected to change with changes in hypertension guidance.^12^ The authors’ estimates of change are therefore likely to be conservative. Results were similar when consultations containing a BP reading alone — where the majority of any misclassification would have occurred — were excluded.

The authors observed different trends in analyses stratified by hypertensive status compared to their main analysis, which included all patients combined. Some patients developed incident hypertension during the study period and, as a result, the main combined analysis includes a greater proportion of patients with hypertension in the later years than in earlier years. Since patients with hypertension also consult more often, this is likely to explain some of the differences seen. Furthermore, this means that the observed, overall downward trend in hypertension-related consultation in this study is likely to be a conservative (under) estimate of true downward trends.

The authors have not considered the clinical content of consultations in finer detail as this would overlap considerably with their previous work.^7^ Finally, interrupted time-series analysis cannot establish causality and these results should be interpreted with caution.

### Comparison with existing literature

The authors’ analyses of hypertension and asthma-related consultations, as well as all consultations, showed similar patterns, suggesting that system-wide changes were influencing all consultation rates during the study period. In this context, the impact of guideline changes will be limited and more difficult to discern in routine data. The authors’ finding that the rate of consultation has fallen over recent years may be surprising given that GPs reported increased workload up to 2017,^13^ and media portrays a service in crisis.^14^ The authors have considered consultation rates using patient person–years as the denominator, and not the number of general practice staff. The number of full-time equivalent GPs fell from 36 069 in 2012 to 33 804 in 2016,^15^ despite increases in the population. Although the number of full-time equivalent nurses increased during this time (from 14 695 to 15 827),^15^ the majority of consultations in this study were conducted by GPs. This may indicate problems with access rather than decreased demand per se. Further work would be required to examine consultation rates using staff numbers as the denominator.

The rate of hypertension-related consultation in patients with hypertension was higher than may be expected given guidance to provide an annual review.^5^ However, nearly two-thirds of patients with hypertension have at least one other condition,^16^ and the authors’ definition is likely to have captured consultations in which an opportunistic BP reading was taken despite hypertension not being the primary reason for consultation. Many GPs may manage hypertension as an add-on problem in this way, and this may explain the high proportion of consultations conducted by a GP in this study. When the authors excluded consultations containing only a BP reading, the rate of hypertension-related consultation was consistent with an annual review.

Changes in hypertension-related workload primarily occurred around the time of diagnosis, suggesting diagnoses are confirmed more quickly and with fewer visits than previously. This is consistent with GP survey data indicating that the majority of practices now offer out-of-office monitoring for diagnosis.^17^ The authors have not provided cost estimates as part of this study, but recent formal economic analyses have shown that out-of-office monitoring is cost saving compared to clinic BP measurement, with savings of up to £186 per person.^18^

### Implications for research and practice

The introduction of the NICE hypertension guideline in 2011 was predicted to reduce workload by reducing the number of false diagnoses in people with white coat hypertension and workload related to subsequent management. The authors have shown that workload related to making a diagnosis more generally has reduced, rather than in the specific context predicted. Indeed, the authors have previously shown that the rate of incident hypertension actually increased after the guideline change, compared to what would have been expected based on previous trends.^7^ Further research would be required to understand why this is the case.

Although the authors cannot establish causality, their findings indicate that the implementation of out-of-office monitoring for diagnosis does not increase general practice workload, and may deliver time savings. Although the vast majority of practices report having access to home or ambulatory BP measurement devices for diagnosis, provision has been shown to vary regionally,^17^ and individual GPs report variable use of self-monitoring for diagnosis.^19^ The authors have previously shown that the use of out-of-office monitoring increased substantially after the change in guidelines, although levels of use remained low compared to clinic BP.^7^ Practices should seek to address equipment shortages where possible and increase awareness of the potential for time savings among individual practitioners.
